# Functional and structural outcomes and complications after pars plana vitrectomy for severe features of proliferative diabetic retinopathy in type 1 and type 2 diabetes mellitus

**DOI:** 10.1371/journal.pone.0288805

**Published:** 2023-07-20

**Authors:** Karolina Kaźmierczak, Paweł Żuchowski, Joanna Stafiej, Grażyna Malukiewicz

**Affiliations:** 1 Department of Ophthalmology, Collegium Medicum in Bydgoszcz, Nicolaus Copernicus University, Toruń, Poland; 2 Clinic of Rheumatology and Connective Tissue Diseases, Jan Biziel University Hospital No. 2, Collegium Medicum in Bydgoszcz, Nicolaus Copernicus University, Toruń, Poland; Yamagata University Faculty of Medicine: Yamagata Daigaku Igakubu Daigakuin Igakukei Kenkyuka, JAPAN

## Abstract

**Purpose:**

To evaluate the functional and structural outcomes as well as postoperative complications after pars plana vitrectomy (PPV) for severe features of proliferative diabetic retinopathy (PDR) in type 1 and type 2 diabetes mellitus (DM) patients.

**Methods:**

Twenty two eyes of type 1 diabetics (DM1 group) and 27 eyes of type 2 diabetics (DM2 group) were included. Best corrected visual acuity (BCVA), intraocular pressure (IOP), postoperative structural changes in optical coherence tomography (OCT) and postoperative complications such as recurrent vitreous haemorrhage, diabetic macular oedema, secondary glaucoma and persistent tractional retinal detachment (TRD) were assessed and compared between the two groups.

**Results:**

Complete reattachment of retina was achieved in 88.9% from the DM1 group and in 95.5% from the DM2 group and remained attached in follow-up. BCVA in DM2 group was significantly lower preoperatively (p = 0.04). Mean postoperative BCVA significantly improved in both studied groups, but it was more evident in eyes of type 2 diabetics compared to type 1 diabetics. In eyes in the DM1 group there was perceptible stabilisation of BCVA. Poor visual acuity or lack of improvement in BCVA in the DM1 group was related to preoperative subretinal haemorrhage in macular region, and TRD involving macula, whereas in the DM2 group–to preoperative subretinal haemorrhage and neovascular glaucoma. The postoperative structural changes (disruption of EZ and ELM) were observed more often in DM2 group, but had the greatest impact on BCVA in eyes of type 1 DM. Complications after PPV for PDR were rare and hadn’t a significant influence on the final functional outcomes in both groups.

**Conclusions:**

Functional improvement after PPV for severe features of proliferative diabetic retinopathy were more noticeable in patients with type 2 DM. Postoperative structural changes had more negative impact on BCVA in type 1 diabetics.

## Introduction

Diabetic retinopathy (DR) is one of the leading causes of blindness in adults in industrialized countries and the emerging cause of blindness in developing countries. The prevalence of diabetes mellitus (DM) in all age-groups worldwide is estimated to be 2.8% in 2000 and 4.4% in 2030 [1]. The number of people with this retinal vascular disease is increasing due to population growth, aging, urbanization, and increasing prevalence of obesity and physical inactivity. DM is also the leading cause of new cases of blindness among adults aged 18–64 years. Among US adults aged 18 years or older with diagnosed DM, crude date for 2019 indicated 11.1–12.4 million reported cases of severe vision difficulties or blindness [2]. Recent estimates suggest that approximately 486 million people worldwide have DM and that roughly one-third demonstrate evidence of DR, including diabetic macular oedema (DME) [3–7]. Hyperglycaemia and molecular events related to DM lead to retinal vessel endothelial cell damage, increased vascular permeability, bleeding, retinal vessel occlusion, and subsequently–retinal ischemia. An ischemic retina secretes locally active cytokines, such as vascular endothelial growth factor and connective tissue growth factor, which lead to neovascularization and connective tissue formation in the proliferative stage of DR. This fibrovascular proliferation grows into the vitreoretinal interface and may contract, potentially resulting in tractional retinal detachment (TRD) [8]. TRD in the setting of proliferative diabetic retinopathy (PDR) represents one of the most advanced stages of DR that often requires pars plana vitrectomy (PPV) to restore vision or prevent further vision loss. PPV plays a crucial role in the management of certain scenarios in DR. These include non-clearing vitreous haemorrhage (VH), TRD and vitreoretinal interface abnormalities impending macular oedema resolution. Numerous reports over the past 40 years have clearly established the beneficial effect of vitrectomy in these settings [9–13]. Moreover, introduction of the anti-VEGF intravitreal injection prior to PPV has significantly improved microsurgery procedures due to intraoperative bleeding reduction and more efficient delamination, thus shortening operation time [14–16]. However, there are still some cases of most advanced stages of PDR where surgical repair remains challenging and the postoperative outcome uncertain. This is specifically true for PDR in patients with type 1 DM. The eyes of such patients are more vulnerable to ischemic insult compared to type 2 diabetics, leading to rapid progression of PDR. Moreover, because of the absence of posterior vitreous detachment, broad-based attachments of severe active fibrovascular proliferation and a higher rate of postoperative complications, PPV is often very demanding in young diabetics [17, 18]. It is believed to be the reason for poorer visual and anatomic outcomes compared with those with type 2 DM [19]. There are few studies comparing severe retinal changes due to progressed PDR in type 1 with type 2 diabetic patients [17–19]. In this study we aim to evaluate the functional and structural outcomes and postoperative complications among type 1 and type 2 diabetics treated with vitrectomy for severe features of proliferative diabetic retinopathy.

## Materials and methods

This was a retrospective comparative observational study of diabetic patients with the most progressed cases of PDR who underwent 23G pars plana vitrectomy at the Ophthalmology Department University Hospital No 1, Collegium Medicum Nicolaus Copernicus University in Bydgoszcz, Poland, from 1^st^ January 2017 to 31^st^ December 2021. The protocol of the study was approved by the Bioethical Commission of Nicolaus Copernicus University, Collegium Medicum in Bydgoszcz (KB 442/2019) and conducted in accordance with the Declaration of Helsinki. We obtained informed verbal consent from each of the participants. Patients were divided into 2 groups: PDR in type 1 diabetes (DM1 group) and PDR in type 2 diabetes (DM2 group). From all patients basic demographic and clinical information was obtained, including diabetic history, previous treatment of DME, laser therapy. Each patient had assessment of Hb1Ac at the time of surgery. The diagnosis of type of DM was based on the patient’s medical history from the patient’s respective diabetes specialist. The history of concurrent systemic diseases (chronic renal disease, ischemic heart disease), as well as ocular disturbances, such as glaucoma, was obtained also from patient’s medical history.

The surgical indications for the study included: PDR with progressive fibrovascular proliferation for all patients, with florid neovascularization, macular TRD or progressive extramacular TRD that threatened the macula or caused distortion in vision. Eyes with coexistent non-clearing dense VH were included if TRD was detectable in preoperative ultrasound examination. The severity of the VH was assessed using Diabetic Retinopathy Vitrectomy Study grading system of the VH (Grade 0 –no VH; Grade I–mild VH with visible fundus details; Grade II–moderate VH with no visible fundus details but with an orange fundus reflex; Grade III–severe VH with no retinal details and no orange fundus reflex). Our clinic’s policy is always doing lens extraction with artificial lens implantation prior to PPV. All studied eyes were pseudophakic, and PPV was performed as the primary vitreoretinal procedure, except for intravitreal anty-VEGF injection for DME treatment. Exclusion surgical criteria were: any other vitreoretinal diseases, surgical procedure of the eye (apart from cataract extraction), and only vitreous haemorrhage as an indication for PPV. Eventually, 49 eyes were enrolled into the study (22 eyes in the DM1 group and 27 eyes in the DM2 group). In the majority of eyes (87.8%), due to massive retinal neovascularization, preoperative intravitreal injection of 0.05ml Ranibizumab was performed 2–4 days prior to surgery. All patients underwent 23G pars plana vitrectomy using Alcon Constellation Vision System or Dorc Eva Phaco-Vitrectomy System which was performed by 2 experienced vitreoretinal surgeons (JS, KK). After a triamcinolone-assisted core vitrectomy, delamination and dissection of the fibrovascular membrane was performed, using different instrumentation, including a high-speed cutter, end-gripping forceps, vertical and horizontal micro scissors, spatula, micro pics and flex loop. Additionally, in some extensive, tightly retina-adhering fibrovascular membranes the use of bimanual technique with chandelier illumination was necessary. In all eyes double staining with Twin Dye (Alchimia srl—M.I.S.S Ophthalmics Ltd) was used for peeling internal limiting membrane (ILM). After removal of the fibrovascular component of ischaemic retina, complete shaving of the vitreous base was performed, followed by fluid-air exchange. All possible breaks found during vitrectomy and 360° peripheral retina were lasered. At the end of the surgery, gas tamponade (C3F8 or SF6) or silicone oil tamponade were used, the latter in cases with residual traction, persistent subretinal fluid, retinotomies and extensive neovascularization with threatening recurrent VH. In eyes with residual traction caused by very stiff retina or fragments of cropped fibrovascular membrane strictly attached to main vessels the use of silicon oil tamponade was necessary.

Postoperative recurrent VH, DME, secondary glaucoma and persistent TRD were defined as complications after PPV. Persistent TRD was also noted as a poor involution of postoperative PDR and was seen in a few of the most severe cases.

All patients underwent clinical examination at minimum 3 months after surgery, which included BCVA assessment (using Snellen charts, decimal fraction), intraocular pressure (IOP), slit-lamp biomicroscopy (with evaluation of presence of abnormal pupil reactions, neovascularization of the iris and iridocorneal angle), indirect ophthalmoscopy and optical coherence tomography (Triton^TM^ Swept Source OCT, Topcon). OCT was used for detection of structural changes of the retina, including ellipsoid zone (EZ) disruption and external limiting membrane (ELM) disruption, intraretinal cystic spaces and persistent subretinal fluid appearance. EZ is most commonly use description for the second hyper-reflective band of the outer retinal layers. In our study every study subject underwent OCT examination with macular thickness analysis using macular cube 512x128 feature. EZ disruption was graded as: Grade 0: Intact photoreceptor EZ; Grade 1: Focal disruption (subfoveal localized involvement) and Grade 2: Global disruption (generalized involvement within the macular cube).

All of the postoperative data (BCVA, IOP, slit-lamp biomicroscopy, indirect ophthalmoscopy and OCT) were collected at the last visit of each patient at our Department.

### Statistical analysis

The data obtained are presented as a mean ± standard deviation (SD). For normally and equally distributed data, the groups were compared using a t-test. For examination of parameter distribution in the study group, a chi-squared test was used. p ≤ 0.05 was considered a statistically significant difference. Statistical analysis was performed using MedCalc® Statistical Software version 20.120 (MedCalc Software Ltd, Ostend, Belgium; https://www.medcalc.org; 2022).

## Results

### Preoperative characteristics

A total of 49 eyes from 40 patients (19 female, 21 male) were enrolled into the study. Twenty two eyes (44.9%) of 18 patients were included in the DM1 group and 27 eyes (55.1%) of 22 patients in the DM2 group. Mean age of patients at the time of surgery was 43.4 (±10.5) years (range 28–64 years) and 65.8 (±6.8) years (range 51–78 years), respectively (p<0.01). Baseline preoperative characteristics for both groups are presented in Table 1.

**Table 1:** Baseline clinical characteristics of patients from DM1 and DM2 groups.

Clinical characteristics	DM1 eyes, n = 22	DM2 eyes, n = 27	p
Patients	18 (45%)	22 (55%)	0.98
Female	10 (55.6%)	9 (40.9%)	0.39
Male	8 (44.4%)	13 (59.1%)	0.41
Right eye	10 (45.5%)	16 (59.3%)	0.34
Left eye	12 (54.5%)	11 (40.7%)	
Mean age at surgery ±SD, years	43.4 ± 10.5	65.8 ± 6.8	< 0.01
Duration of diabetes ±SD, years	22 ± 9	18 ± 9.3	0.13
Mean follow-up (months) ±SD	8.5 ± 7.6	10 ± 10.4	0.36
Mean preoperative Hb1Ac level (±SD) (range)	7.5± 1.4 (5.4–10.3)	7.1±1.2 (5.7–9.9)	0.21
Chronic kidney disease	3 (13.6%)	0	0.05
Ischeamic heart disease	0	3 (11.1%)	0.11
Preoperative characteristics (eyes)			
Open-angle glaucoma n(%)	2 (9%)	10 (37%)	0.03
Neovascular glaucoma n(%)	0	4 (14.8%)	0.06
Diabetic macular edema n(%)	14 (63.6%)	14 (51.8%)	0.41
Ranibizumab for DME treatment n(%)	4 (18.2%)	4 (14.8)	0.75
Panretinal photocoagulation n(%)	13 (59.1%)	17 (62.9%)	0.78
Tractional retinal detachment n(%)	18 (81.8%)	22 (81.5%)	0.52
Vitreous haemorrhage n(%)	17 (77.3%)	21 (77.8%)	0.97
Subretinal haemorrhage n(%)	6 (27.3%)	3 (11.1%)	0.49
Mean preoperative BCVA±SD Decimal (Snellen)	0.16 ± 0.23	0.04 ± 0.13	0.04
<0.05 (<6/120)	12 (54.5%)	24 (88.9%)	0.06
0.05–0.1 (6/120–6/60)	4 (18.2%)	1 (3.7%)	
0.2–0.5 (6/30–6/12)	4 (18.2%)	1 (3.7%)	
>0.5 (>6/12)	2 (9.1%)	1 (3.7%)	
Mean preoperative IOP (mmHg) ±SD	14.7 ± 3.0	16.2 ± 6.3	0.36

P-value ≤0.05 was considered to be statistically significant

Abbreviations: DM, diabetes mellitus; DME, diabetic macula oedema; BCVA, best corrected visual acuity; IOP, intraocular pressure

Mean duration of diabetes was 22 (±9) years (range 4–40 years) for the DM1 group and 18 (±9.3) years (range 1–30 years) for the DM2 group (p = 0.13). Mean follow-up period after surgery was 8.5 (±7.6) months and 10 (±10.4) months, respectively (p = 0.36). Mean glycated haemoglobin (Hb1Ac) level at the time of surgery was comparable between the two groups (p = 0.21). Chronic renal disease was diagnosed in only 3 patients with type 1 DM (p = 0.05) and none in DM2 group, although we didn’t performed detailed laboratory tests, but relied only on accessible medical history of patients. Before surgery, 51.9% eyes in the DM2 group were diagnosed with glaucoma and in 37% of them it was primary open-angle glaucoma (POAG), with more frequent occurrence than in type 1 diabetics, (p = 0.03). All of these eyes were treated with anti-glaucomatous eyedrops, with good IOP control during the whole follow-up period. Only 14.8% eyes from the DM2 group were diagnosed with neovascular glaucoma (NVG) before surgical intervention, whereas none of the patients from the DM1 group had symptoms of neovascularization in the anterior part of the eye. Eyes with NVG had elevated IOP before surgery regardless of the anti-glaucomatous topical treatment and panretinal photocoagulation (PRP) done in 3 of them. Generally, mean preoperative IOP was slightly higher in the DM2 group (16.1 mmHg vel 14.7 mmHg in the DM1 group), but without statistical significance.

There was noticeable difference in mean preoperative BCVA in the studied groups. Eyes of type 2 diabetics had significantly lower baseline visual acuity compared to those of type 1 DM (p = 0.04). Hand motion was noted in 8 (36.4%) eyes and in 14 (51.9%) eyes from the DM1 group and the DM2 group, respectively. Preoperative light perception was detected in 1 eye from each group.

Based on patients’ medical history, PRP as an initial treatment for PDR was performed in a total of 30 eyes. Preoperative DME was diagnosed in 14 eyes in each group, but only 4 eyes in each were treated with intravitreal injection of Ranibizumab.

A vitreous haemorrhage was observed in approximately 77% of the eyes in both groups, and a subretinal haemorrhage was detected twice more frequently in DM1 group than DM2 group. Dense VH (Grade III) was detected in most eyes from both groups (in 53% eyes from DM1 group and 76% eyes from DM2 group). Grade I and Grade II of VH were observed equally in 22,5% eyes in type 1 diabetics, whereas in DM2 group Grade II was noticed a little more frequently than Grade I (14% vel 10% eyes).

The most common feature of PDR in both groups was TRD, which was observed in more than 80% eyes. In half of the eyes of type 1 diabetics and almost 45% eyes of type 2 diabetes patients retinal detachment comprised the macular region. There was no significant difference between the studied groups regarding TRD involving macula or any of the four quadrants of the retina, although PDR in eyes from the DM1 group tended toward covering more than 2 quadrants of the retina, apart from the macular region. A degree of tractional retinal detachment involvement in both studied groups is featured in Table 2.

**Table 2:** Degree of tractional retinal detachment involvement in DM1 and DM2 groups.

	DM1 eyes, n = 22	DM2 eyes, n = 27	p
Tractional retinal detachment n(%)	18 (81.8%)	22 (81.5%)	0.52
TRD involving macula	12 (54.5%)	12 (44.4%)	0.49
TRD involving superior quadrant	10 (45.5%)	16 (59.3%)	0.34
TRD involving temporal quadrant	12 (54.5%)	8 (29.6%)	0.08
TRD involving inferior quadrant	13 (59.1%)	10 (37%)	0.13
TRD involving nasal quadrant	11 (50%)	10 (37%)	0.37
Macula + 1 quadrant	2 (9.1%)	6 (22.2%)	0.17
Macula + 2 quadrants	2 (9.1%)	3 (11.1%)	0.82
Macula + 3 quadrants	2 (9.1%)	1 (3.7%)	0.44
Macula + 4 quadrants	6 (27.3%)	2 (7.4%)	0.06

P-value ≤0.05 was considered to be statistically significant

Abbreviations: DM, diabetes mellitus; TRD, tractional retinal detachment

### Surgical procedure features

In total, 41 (83.7%) eyes with massive neovascular component of fibrovascular membrane received intravitreal Ranibizumab injection prior to surgical procedure (19 (86.4%) eyes from the DM1 group and 22 (81.5%) eyes from the DM2 group). In all eyes from both groups complete removal of the epiretinal membranes and ILM peeling from the macular region was performed. In 2 eyes (7.4%) from the DM2 group subretinal membranes were also expurgated. Due to the rigidity of the tractional detached retina, retinotomy was necessary in a total of 5 (10.2%) eyes, without significance in either group. Bimanual technique with chandelier illumination usage was required in 6 (27.3%) eyes from the DM1 group and in 11 (40.7%) eyes from the DM2 group (p = 0,33). Gas endotamponade was most commonly used in eyes from the DM2 group (in 17 (63%) eyes), whereas in the DM1 group the same number of eyes received gas tamponade and silicone oil tamponade. Mean time of surgery was similar in both groups, 151±52 minutes and 144±53 minutes for the DM1 group and DM2 group, respectively.

Silicone oil was removed mean 18 months (range 5–33 months) after primary procedure in 7/11 eyes from the DM2 group and mean 10 months (range 6–18 months) in 5/10 eyes from the DM1 group. Surgical procedure characteristics are shown in Table 3.

**Table 3:** Surgical procedure characteristics in eyes from DM1 and DM2 groups.

Surgical characteristics	DM1 group, n = 22 eyes	DM2 group, n = 27 eyes	p
Ranibizumab prior to surgery n(%)	19 (86.4%)	22 (81.5%)	0.55
Retinotomy n(%)	2 (9.09%)	3 (11.1%)	0.82
Chandelier illumination n(%)	6 (27.3%)	11 (40.7%)	0.33
Subretinal membrane removal n(%)	0	2 (7.4%)	0.20
C3F8 gas endotamponade n(%)	11 (50%)	16 (59.3%)	0.52
SF6 gas endotamponade n(%)	0	1 (3.7%)	0.37
Silicone oil endotamponade n(%)	11 (50%)	10 (37%)	0.37
Duration of surgery (minutes) ± SD mean(range)	151 ± 52 (70–260)	144 ± 53 (75–260)	0.65

P-value ≤0.05 was considered to be statistically significant

Abbreviations: DM, diabetes mellitus

### Functional outcomes

Mean postoperative BCVA significantly improved in both studied groups, from 0.16 ± 0.23 to 0.29 ± 0.3 in the DM1 group (p = 0,01) and from 0.04 ± 0.13 to 0.18 ± 0.2 in the DM2 group (p = 0,05). Mean postoperative IOP significantly increased in eyes from the DM1 group (p = 0,04) but was stable in eyes from the DM2 group. The type of tamponade used during vitrectomy had no influence on the final IOP measurements. Mean postoperative change of BCVA and IOP was featured in Table 4.

**Table 4:** Mean postoperative change of BCVA and IOP.

	Before surgery	After surgery	p
Mean BCVA change DM1	0.16 ± 0.23	0.29 ± 0.3	0.01
Mean BCVA change DM2	0.04 ± 0.13	0.18 ± 0.2	0.05
IOP change DM1	14.8 ± 3.0	18.4 ± 7.6	0.04
IOP change DM2	16.1 ± 6.3	16.9 ± 6.1	0.57
Mean IOP change (gas) DM1	15.0 ± 0.9	18.7 ± 9.3	0.20
Mean IOP change (gas) DM2	16.4 ± 7.7	15.8 ± 5.9	0.75
Mean IOP change (silicone oil) DM1	14.5 ± 3.2	18.0 ± 5.8	0.10
Mean IOP change (silicone oil) DM2	15.7 ± 3.3	18.7 ± 6.3	0.08

P-value ≤0.05 was considered to be statistically significant

Abbreviations: DM, diabetes mellitus; BCVA, best corrected visual acuity; IOP, intraocular pressure

Before surgery, the majority (88.9%) of eyes from the DM2 group had poor visual acuity, BCVA < 0.05 (< 6/120). It improved substantially after operation (p = 0,05), with poor vision persisting in only 33% eyes in final control. Hand motion was the lowest postoperative VA, registered in 3 (11.1%) eyes with preoperative subretinal haemorrhage in the macular region. On the other hand, only 1 (3.7%) eye achieved very good visual acuity. Generally, in this group improvement in BCVA was noted in 23 (85.2%) eyes and was stable in 2 (7.4%) eyes, both of them with NVG. Only in the remaining 2 (7.4%) eyes visual acuity worsened, in one of them because of postoperative DME. Eyes from the DM1 group had better preoperative visual acuity, with poor BCVA in only approximately 50% of the eyes, but after surgery 40.9% of the eyes had BCVA on this level still. Analogously to the DM2 group, in 2 eyes with subretinal haemorrhage in macula before surgical intervention VA had not improved and remained on hand motion level. On the other hand, very good BCVA >0.5 (>6/12) was noted in more eyes (18.2%) than in the DM2 group, although it was not confirmed statistically. There was significant correlation between BCVA and attachment of the macula before surgery in eyes from the DM1 group. Those with preoperatively detached macula had worse postoperative BCVA compared to eyes with the macula attached (p = 0,02). This correlation was not evident in eyes from the DM2 group. Generally, in 14 (63.6%) eyes BCVA improved and in 1 (4.5%) eye slightly worsened in the DM1 group. Stabilization of BCVA was achieved in 7 (31.8%) eyes, compared to 2 (7.4%) eyes from the DM2 group (p = 0,03).

Postoperative functional outcomes in final follow-up control are featured in Table 5.

**Table 5:** Postoperative functional outcomes in final follow-up control.

Functional characteristics	DM1 group, n = 22 eyes	DM2 group, n = 27 eyes	p
Mean postoperative BCVA±SD Decimal (Snellen)	0.29 ± 0.3	0.18 ± 0.2	0.16
<0.05 (<6/120)	9 (40.9%)	9 (33.3%)	0.24
0.05–0.1 (6/120–6/60)	3 (13.6%)	8 (29.6%)	
0.2–0.5 (6/30–6/12)	6 (27.3%)	9 (33.3%)	
>0.5 (>6/12)	4 (18.2%)	1 (3.7%)	
Improved n(%)	14 (63.6%)	23 (85.2%)	0.08
Stable (eyes) n(%)	7 (31.8%)	2 (7.4%)	0.03
Worsened (eyes) n(%)	1 (4.5%)	2 (7.4%)	0.68
Mean postoperative IOP (mmHg) ±SD	18.4 ± 7.6	16.9 ± 6.1	0.45
Mean postoperative IOP in eyes with gas endotamponade, mmHg ± SD	18.6 ± 8.9	15.8 ± 6.1	0.33
Mean postoperative IOP in eyes with silicone oil endotamponade, mmHg ± SD	18.1 ± 6.1	18.5 ± 6.0	0.87

P-value ≤0.05 was considered to be statistically significant

Abbreviations: DM, diabetes mellitus; BCVA, best corrected visual acuity; IOP, intraocular pressure

### Structural outcomes

At the final postoperative control mean central retinal thickness in the macular region was comparable in both groups, which was shown in Table 6.

**Table 6:** Postoperative structural outcomes in final follow-up control.

OCT characteristics n(%)	DM1 group, n = 22 eyes	DM2 group, n = 27 eyes	p
EZ disruption	11 (50%)	22 (81.5%)	0.02
ELM disruption	7 (31.8%)	14 (51.8%)	0.16
Persistent subretinal fluid	2 (9.1%)	3 (11.1%)	0.82
Intraretinal cystic spaces	1 (4.5%)	4 (14.8%)	0.24
Mean central retinal thickness ± SD (μm), range	382.5 ± 104 (233–570)	387.9 ± 106 (234–648)	0.86

P-value ≤0.05 was considered to be statistically significant

Abbreviations: DM, diabetes mellitus; OCT, optical coherence tomography, EZ–ellipsoid zone; ELM–external limiting membrane

The intraretinal cystic spaces which indicate at DME were detected only in 1 (4.5%) eye from the DM1 group and 4 (14.8%) eyes from the DM2 group, but on account of the low sample size we were unable to assess their correlation with poor visual acuity. The most prominent change in OCT imaging was EZ disruption, which was present in the majority of eyes from the DM2 group (22 (81.5%) eyes). By comparison, this kind of OCT change was shown only in 50% of the eyes from the DM1 group (p = 0,02). However, in the DM1 group, EZ disruption was the reason for significantly lower BCVA in postoperative follow-up (p = 0,04). Using grading of EZ disruption, we found out, that in type 1 diabetics grade 2 was seen more often. ELM disruption was observed less often, in 14 (51.8%) eyes from the DM2 group and in 7 (31.8%) eyes from the DM1 group and had no significant correlation with BCVA. Persistent subretinal fluid in the macular region was detected in 2 (9.1%) eyes from the DM1 group and in 3 (11.1%) eyes from the DM2 group, which resulted in lack of improvement in BCVA, although it wasn’t statistically significant.

### Postoperative complications

A good involution of PDR was noted in the majority of studied eyes in both groups. Reattachment of the retina was achieved in 16/18 eyes (88.9%) from the DM1 group and in 21/22 eyes (95.5%) from the DM2 group. Involution of PDR and postoperative complications in follow-up are presented in Table 7.

**Table 7:** Involution of PDR and postoperative complications in follow-up.

Clinical features	DM1 group, n = 22 eyes	DM2 group, n = 27 eyes	p
Good involution of PDR n(%)	20 (91%)	26 (96.3%)	0.19
Postoperative complications n(%)			
Vitreous haemorrhage	2 (9.09%)	4 (14.8%)	0.55
Persistent TRD	2 (9.09%)	1 (3.7%)	0.44
Persistent DME	1 (4.5%)	4 (14.8%)	0.24
Secondary glaucoma	3 (13.6%)	3 (11.1%)	0.79

P-value ≤0.05 was considered to be statistically significant

Abbreviations: DM, diabetes mellitus; PDR, proliferative diabetic retinopathy; TRD, tractional retinal detachment; DME, diabetic macula oedema

In the 3 remaining eyes with TRD and preoperative subretinal haemorrhage in the macula region with submacular scarring (2/18 (11.1%) eyes from the DM1 group and in 1/22 (4.5%) eye from the DM2 group) retinal detachment was progressed to such a degree that complete reattachment of the retina was not possible. Because of the high degree of ischaemia and the destruction of the retina in the macular region, we decided not to continue with surgical treatment. In these eyes visual acuity did not improve in the postoperative period. Apart from these, in all the other cases of TRD retina was attached during primary procedure and remained attached in follow-up. Any secondary surgical management in these eyes involved silicone oil removal which was performed in 7 (63.6%) eyes in the DM1 group and in 5 (50%) eyes from the DM2 group without any complications.

Recurrent VH appeared in 2 (9.09%) eyes and 4 (14.8%) eyes, respectively. However, it disappeared spontaneously sometime in the next few months without negative influence on the final visual acuity in either group. Persistent DME was observed in 1 (4.5%) eye from the DM1 group and in 4 (14.8%) eyes from the DM2 group and was also the reason for weak improvement in postoperative BCVA. The poor involution of PDR wasn’t correlated with level of Hb1Ac or duration of diabetes in either of studied groups.

Secondary glaucoma was detected in 3 eyes in each group. All eyes from the DM2 group and 2 from 3 eyes from the DM1 group received silicone oil tamponade, but again, on account of the low sample size we were unable to assess their correlation with poor visual acuity.

## Discussion

It is estimated that in Europe there are 59 million diabetic patients, and 2.34 million adults are diagnosed with DM in Poland alone [20–22]. Where type 2 DM amounts to 85%–95% of diabetes cases with a significant expansion of new cases, type 1 DM constitutes 15%–20% of the diabetic population and is found particularly often in Europeans [23–26]. The incidence rate of type 1 DM in Poland is 10.2/100,000 people/year with an increasing frequency, and the probability of developing ocular complications in the course of diabetes should be expected more frequently [27]. A global estimate of the overall prevalence of DR and PDR is 34.6% and 7%, respectively [4]. Matuszewski et al. estimated the prevalence of DR in North-East Poland, which could be extrapolated to the whole country, overall 25.48% (32.58% for type 1 DM and 23.04% type 2 DM), whereas the prevalence for PDR was 1.59% and 1.01%, respectively [28]. It is estimated that 25% of DR-related vision loss stems from PDR complications, such as VH, TRD and severe fibrovascular proliferation [29, 30]. These complications require surgical management to prevent complete damage of the retina. Pars plana vitrectomy was introduced in vitreoretinal surgery 50 years ago and was successfully performed in different aspects of PDR [31, 32]. Thanks to advances in surgical techniques, from small-gauge vitrectomy system and ancillary instrumentation essential to safe and complete removal of the fibrovascular tissue from retinal surface, more indications for PPV were possible in severe cases of PDR [33–35]. Nevertheless, TRD remains the primary reason for PPV and constitutes 40% of diabetic vitrectomies [30, 36]. It is believed that patients with type 1 DM are particularly susceptible to severe PDR complications because of long duration of diabetes and specific anatomical circumstances, such as broad vitreoretinal adhesion and severe active fibrovascular proliferation [17, 18, 37]. The visual outcomes and complications in vitrectomized eyes were commonly analysed in older diabetics or generally in patients with DM without categorization by type [38–43]. However, there are few studies in young diabetics and few publications which compared functional and structural results in type 1 and type 2 DM, especially in the most-progressed cases of PDR [17–19]. We compared both visual and anatomical postoperative results after PPV in the most-progressed cases of PDR between type 1 and type 2 DM. The basic demographic characteristics were similar to those found in literature. Patients with type 1 DM were definitely younger, with a longer duration of diabetes, however the length of the disease in that group wasn’t significantly dissimilar to that of type 2 DM patients. Previous studies have shown that prevalence rates for PDR are substantially higher in patients with type 1 diabetes with a longer duration of DM, higher HbA1c, and elevated blood pressure as risk factors [4]. In our study, although the preoperative main level of Hb1Ac was slightly higher in patients from the DM1 group compared to the DM 2 group, it was still in the lower ranges compared to other studies [20, 21]. It could be related to the preoperative preparation of patient, with a rigorous treatment of systemic diseases and good glycaemic control during operative time.

The course of diabetes mellitus may rapidly change with progression of diabetic complications if other general disorders, namely systolic hypertension, hyperlipidaemia, obesity or microalbuminuria, occur. It was reported that the prevalence of chronic kidney disease is higher in type 1 DM patients and young diabetics [17, 19]. In patients requiring vitrectomy, renal compromise may be a reason for higher risk of intraoperative and postoperative bleeding, delayed wound healing and an increase in mortality [44]. In our study 3 patients from the DM1 group was diagnosed with chronic renal disease, whereas non from DM2 group. However, we can’t have a definite certainty that there haven’t been cases of undiagnosed microalbuminuria or lower level of eGFR in some of the studied patients. Because of the small simple size of patients with diagnosed chronic renal disease and unsurety about microalbuminuria status we’re not able to determine the connection between this general disorder and PDR progression.

Association between diabetes mellitus and glaucoma has been a matter of debate in the past, but recently it was established that the prevalence of glaucoma is approximately two to three times higher in the diabetic population compared to the nondiabetic one [45–48]. We’ve found that incidence of preoperative POAG was higher in eyes from the DM2 group compared to those from the DM1 group, although the level of IOP was stable before and after vitrectomy. On the other hand, there was a significant increase in postoperative IOP in the DM1 group, but few eyes required anti-glaucomatous treatment (only 1 patient was diagnosed with secondary glaucoma and needed anti-glaucomatous eye drops). The type of tamponade hasn’t significantly influenced postoperative measurements. The correlation between elevation of IOP in diabetic patients and silicone oil tamponade is still controversial. Some authors claim that IOP rise after silicone oil injection is independent from diabetes, while others suggest that patients with DM are at a lower risk to develop an elevation of IOP than non-diabetic ones [49, 50]. On the other hand, many studies support the correlation between diabetes and secondary glaucoma due to silicone oil tamponade [51–53]. It is believed that in the early postoperative period, the IOP elevation can be secondary to an overfill of silicone oil, a pupillary block, the migration of silicone oil into the anterior chamber, postoperative inflammation and/or steroid-induced ocular hypertension [54]. In the late postoperative period, IOP elevation may occur due to a pupillary block, synechial angle closure, rubeosis iridis and migration of emulsified silicone oil into the anterior chamber [55]. We’ve observed no emulsification of silicone oil, migration of silicone oil to the anterior chamber or pupillary block in any patient during the postoperative period, although the follow-up of our study is quite short and we need to continue observation of these patients. Our findings also have to be consider with caution, because we’ve studied very specific group of diabetic patients–with most progressed, complicated cases of PDR, therefore we can’t draw a conclusion for the whole diabetic population.

According to literature, NVG accounts for approximately 5% of blindness in diabetics. In the case of NVG in one eye of a DM patient, there is a relatively high risk of NVG development in the fellow eye without prophylactic treatment [56]. Liao M et al. reported a higher incidence of NVG in young diabetics compared to senior [17]. Their findings are dissimilar to ours, because we detected NVG in 4 eyes from the DM2 group (all more than 55 years old) yet none in the DM1 group. Although the incidence of NVG wasn’t statistically significant, it implies that progressed ischaemia could be seen in older diabetics as well. Sakamoto et al. established risk factors for developing NVG in vitrectomized eyes. These were preoperative parameters such as elevated IOP, iris/angle neovascularization, low HbA1c level and administration of insulin, as well as use of retinal tamponade during retinal surgery [57]. All 4 eyes from the DM2 group with detected NVG in our study had every one of these risk factors. Therefore, our findings are consistent with Sakamoto’s results.

Thanks to advancements in vitrectomy in recent years, the rates of primary reattachment and final attachment of retina after vitrectomy for TRD have increased, ranging from 86.3 to 100% [18, 19, 32, 40, 43]. Primary reattachment of retina in our patients was achieved in 88.9% and 95.5% of the eyes from the DM1 and DM2 groups, respectively. During follow-up, no patient from either group had secondary retinal detachment. This is in contrast to other authors who have reported reoperation necessity due to recurrent TRD in 13.2–36% [18, 19, 40]. Notably, further surgery was required more often in eyes of younger diabetics with type 1 DM. Huang et al. assessed the recurrent retinal detachment rates as 13.2% for younger diabetics and 1.3% for older (>40 years old) [18]. A recent study of Kumar et al. has shown a need for reoperation of TRD in 23.8% of the patients with type 1 DM and in 15.5% of the patients with type 2 DM [19]. Author’s explanation for the lower percentage of final reattachment and higher rates for needed secondary surgery in eyes of type 1 diabetics was a higher proportion of complex detachments with macular involvement in this group. With 3 eyes from our study (2 out of 18 (11.1%) eyes from the DM1 group and 1 out of 22 (4.5%) eyes from the DM2 group) we failed in achieving a reattachment of retina after primary procedure because of severe ischaemia and degree of destruction in the macular area. Due to a poor prognosis of reoperation, we excluded these patients from further surgeries. We suspect that the primary medical assessment and clinical qualification for surgery in these eyes were incorrect due to poor prognosis for recovery and vision gain. On the other hand, we hadn’t observed redetachment of retina in any patient with primary surgical success in either group in follow-up. During our operations we strived to remove all of the subretinal and epiretinal fibrotic tissue, which we believe was possible due to intravitreous injection of VEGF inhibitor a few days prior to surgery. It was reported that preoperative Bevacizumab decreases intraoperative bleeding and postoperative VH but also simplifies surgery due to easier delamination of complex fibrovascular membranes [14, 15, 58, 59]. Most of our patients received a preoperative injection of Ranibizumab. We hadn’t observed intensive bleeding during surgical procedure or significant recurrent postoperative VH. In our opinion, dissection and delamination of the fibrovascular membranes were much easier to perform and sometimes, in cases with very extensive neovascularization, only possible thanks to intravitreal injection of this drug. We also used more frequently than in most other studies silicone oil tamponade, which in our opinion enabled us to perform complete PRK in the postoperative period and consequently protect the eye from recurrent VH and redetachment of retina. The necessity of silicone oil tamponade in our patients’ eyes was higher than in the Huang et al. study (38.2%) but lower than in the Kumar et al. study in both diabetic groups (85.7% and 87.9% for type 1 DM and type 2 DM, respectively) [18, 19].

It is reported that about 30% of patients develop postoperative recurrent VH from vitreous base neovascularization, neovascularization near sclerotomies, or diffusion from vitreous blood clots in peripheral retina [30, 60]. The prevalence of recurrent postoperative VH in our study was lower than in other publications, perhaps due to the fact that we’ve routinely lasered the peripheral retina at the vitreous base during every operation and completed PRP soon after the surgical procedure. Although there was no statistical significance, VH appeared two times more frequently in eyes of the DM2 group compared to the DM1 group. All of recurrent postoperative vitreous haemorrhage which appeared in 2 (9.09%) eyes from the DM1 group and in 4 (14.8%) eyes from the DM2 group was treated in primary surgery with usage of gas tamponade and disappeared spontaneously after a few months.

Advantages of internal limiting membrane (ILM) peeling during vitrectomy for PDR are well known. Removing ILM guarantees the elimination of adherent cortical vitreous and therefore decreases the possibility of epiretinal membrane formation and macular oedema, as well as ensures complete eradication of multiple membranes in complex TRD [19, 61, 62]. We’ve made similar observations as ILM peeling was performed in all operated eyes, even without TRD in macular region. Although, we don’t have statistical support, we strongly believe that routine ILM peeling during vitrectomy in progressed cases of PDR may protect the retina from secondary fibrotic tissue formation and diabetic macular oedema.

Most studies show that functional outcomes improve after vitrectomy for PDR in 49–83% eyes and with current surgical technique progression to no light perception is unusual [30, 32, 34, 42, 63–66]. Liao et al. compared postoperative changes in BCVA between young diabetics and senior ones [17]. They observed improvement in 70.7% and 81% eyes, stabilization in 15.5% and 3.4% eyes, and decrease in 13.8% and 15.5% eyes, respectively. They concluded that young patients had more eyes with low vision either before surgery, after surgery and at a final follow-up. Kumar et al. in a similar study reported clinical features and surgical outcomes after vitrectomy in type 1 and type 2 diabetics. They observed improvement in VA in 61.9% and 75.7% eyes, stabilization in 11.9% and 8.6% eyes, and decrease in 21.4% and 6.9% eyes, respectively [19]. Very good postoperative VA (≥20/40) was achieved in 14.3% eyes of type 1 diabetics and 24.1% eyes of type 2 diabetics. BCVA of 20/200 had a similar number of eyes from both groups (52.4% and 55.2%, respectively), and ambulatory vision (≥20/400) gained 66.7% and 79.3% eyes, respectively. They also drew conclusions that visual outcomes were poorer in patients with type 1 DM. In our study eyes of type 2 DM patients had preoperatively lower VA than those of type 1 DM, but just as in the other authors’ findings, BCVA significantly improved after surgery in both groups. Comparing the change in visual acuity between both groups, improvement in BCVA was noted in 63.6% and 85.2% eyes, stabilization in 31.8% and 7.4% eyes, and decrease in 4.5% and 7.4% eyes for the DM1 and DM 2 groups, respectively. Similarly to others authors, improvement in VA was more expressed in type 2 diabetics compared to type 1 ones. On the other hand, in contrast to other researchers, we observed a greater number of eyes from the DM1 group with stabilization of VA than decrease.

Studies show a tendency for visual acuity to change during the initial 6 months after surgery for PDR and stabilisation at 1 year [67]. The initial instability was related to preoperative retinal detachment. As predictors for poor visual outcomes are usually quoted iris rubeosis and NVG, vitreopapillary traction, poor preoperative visual acuity and presence of TRD, especially in the macular region [18, 30, 68–70]. Postoperative macular ischemia, VH and iris rubeosis are acknowledged as the most important correlates for poor VA following vitrectomy for PDR [69, 71]. Our findings are consistent with other reports, although distribution of some risk factors, such as NVG in type 1 and type 2 DM, was dissimilar. In summary, poor visual acuity and lack of improvement in BCVA in DM1 patients was connected with preoperative subretinal haemorrhage in the macular region and TRD involving macula, whereas in the DM2 group–with preoperative subretinal haemorrhage and NVG.

The introduction of OCT into diagnostics of various retinal diseases, including diabetic retinopathy, enabled detection of subtle changes in the macular structure in the course of the disease before and after vitrectomy [72–77]. One of the common OCT findings after clinically successful vitrectomy for TRD is persistent subretinal fluid. It resolves slowly over time, with gradual improvement in visual acuity. Algethami et al. in retrospective interventional case series of 46 eyes reported prevalence of persistent subretinal fluid in 100% eyes at 3 months and in 23.8% eyes at 12 months after surgical intervention, but only in 6.5% eyes at final follow-up [77]. The authors concluded that additional drainage of the subretinal fluid may not be necessary. On the other hand, Karimov et al. in a study of residual subfoveal fluid after successful PPV in patients with diabetic tractional macular detachment observed that non-drainage of the fluid is a significant risk factor for long-standing persistent subretinal fluid, although they mentioned that internal drainage may be performed if intraoperative breaks are found. They reported the prevalence of subretinal fluid in 91.7% eyes at 3 months and 4.2% eyes at 12 months after surgery [78]. In our study persistent subretinal fluid in the macular region was detected in 2 (9.1%) eyes from the DM1 group and in 3 (11.1%) eyes from the DM2 group, which resulted in lack of improvement in BCVA. None of the eyes had intraoperative drainage of subretinal fluid. We noticed different observation times for these groups. The follow-up for 3 eyes with subretinal fluid of patients from the DM2 group was 3 months, whereas for 2 eyes from the DM1 group it was 12 months. Although we’re unable to determine how quickly subretinal fluid will resolve in both groups, our findings may suggest that in eyes of type 1 diabetics resolution of subretinal fluid proceeds slowly.

Despite the fact that, because of retinal non-perfusion, diabetic retinopathy is classified as an inner retinal disease, some structural changes in the outer retina may be observed as epiretinal and subretinal fibrosis and traction unfold [72]. The two innermost bands in the outer retina (ELM and EZ) are considered to represent photoreceptors integrity and their disruptions correlate with poor visual acuity in PDR [72–74, 76]. In our study the EZ disruptions in OCT scans were observed more often in eyes from the DM2 group (81.5% eyes) compared to eyes from the DM1 group (50%) (p = 0,02). However, the EZ disruption had the greatest impact on BCVA in eyes of type 1 DM (p = 0,04). ELM disruption was observed less often in both groups and had no significant correlation with BCVA.

It was reported that central point thickness has no significant influence on BCVA or correlate weakly with postoperative visual acuity [73, 74]. Dooley et al. affirmed that preoperative and postoperative DME, defined as any cystoid spaces in SD-OCT images, does not correlate with final BCVA [72]. Our observations are consistent with reports of other authors as we found no correlation between postoperative central retinal thickness or postoperative DME and visual acuity in either group. We agree with Shah et al. that outer retinal microstructure might be a better anatomical representation of postoperative function than retinal thickness in eyes after PPV for fibrovascular complications of PDR [74].

In summary, a reattachment of retina in our patients was achieved in 88.9% eyes from the DM1 group and in 95.5% eyes from the DM2 group and remain attached during follow-up. Functional improvement after PPV for severe features of proliferative diabetic retinopathy were more noticeable in patients with type 2 DM. Postoperative structural changes had more negative impact on BCVA in type 1 diabetics. The complications after PPV for PDR were rare and had no significant influence on the final functional outcomes in both groups.

We are aware of several limitations in our study, such as the absence of randomization, relatively short and varied time of follow-up and small sample size. Additionally, because of a variety of the preoperative PDR changes in our patients with different postoperative prognosis, regardless of DM type, our findings may only provide general information due to the sample size that limited further categorization.

Further studies in larger patient samples are needed for more accurate validation of our findings in functional and structural outcomes after vitrectomy for progressed stage of PDR in eyes with type 1 compared to type 2 diabetics.

## Supporting information

S1 Data (ODS)
